# THE ASSOCIATION OF A NOVEL PHYSICAL ACTIVITY METRIC WITH COGNITION AND RISK OF DEMENTIA

**DOI:** 10.1093/geroni/igad104.1296

**Published:** 2023-12-21

**Authors:** Yurun Cai, Junhong Zhou, Qu Tian, Amal Wanigatunga, Eleanor Simonsick, Susan Resnick, Luigi Ferrucci, Jennifer Schrack

**Affiliations:** University of Pittsburgh, Pittsburgh, Pennsylvania, United States; Harvard Medical School/Hebrew SeniorLife, Roslindale, Massachusetts, United States; National Institute on Aging, National Institutes of Health, Baltimore, Maryland, United States; Johns Hopkins School of Public Health, Baltimore, Maryland, United States; National Institute on Aging, National Institutes of Health, Baltimore, Maryland, United States; National Institute on Aging, National Institutes of Health, Baltimore, Maryland, United States; National Institute on Aging, National Institutes of Health, Baltimore, Maryland, United States; Johns Hopkins University, Baltimore, Maryland, United States

## Abstract

Physical activity (PA) is associated with cognitive function and Alzheimer’s disease and related dementias (ADRD). Although associations between continuously measured PA and ADRD have been assessed, multiscale entropy (MSE), which quantifies dynamics of physiological systems over multiple time scales, has not been used to quantify daily activity rhythms in older adults by cognitive status. We examined the association of this novel metric of PA complexity with cognitive function, mild cognitive impairment (MCI), and dementia in participants of the Baltimore Longitudinal Study of Aging. A total of 615 older adults (mean age=73.9±11.3 years, 54.5% women) completed a 7-day wrist-worn accelerometer assessment between 2015-2019. Global cognitive function was measured using the Mini-Mental State Examination (MMSE). Mild cognitive impairment (MCI) or dementia was diagnosed based on Petersen criteria and Diagnostic and Statistical Manual of Mental Disorders, respectively. Unadjusted logistic regression models showed that participants in the lowest tertile of complexity had 2.31 times the odds of low MMSE score (≤26) compared to those in the highest tertile (odds ratio [OR]=2.31, 95% confidence interval [CI]=1.09-4.89). This association lost significance after adjusting for age, sex, race, and education years. The lowest tertile of complexity was also associated with 2.58 times the odds of MCI/dementia diagnosis, adjusting for demographics (OR=2.58, 95% CI=1.06-6.31). These results suggest that lower complexity of accelerometry-detected movement is associated with poorer cognitive function and greater risk of MCI/dementia. Future longitudinal studies are warranted to examine whether altered complexity of daily activity rhythms may act as a preclinical indicator of ADRD.

